# Effects of rocuronium and vecuronium on initial rundown of endplate potentials in the isolated phrenic nerve diaphragm preparation of rats

**DOI:** 10.1186/2193-1801-2-155

**Published:** 2013-04-11

**Authors:** Jun Li, Yong-Qin Liu, Han-Ting Zhang

**Affiliations:** 1 Department of Anesthesiology, Navy General Hospital of PLA, Beijing, 100037 China; 2 Departments of Behavioral Medicine & Psychiatry and Physiology & Pharmacology, West Virginia University Health Sciences Center, Morgantown, WV 26506 USA

**Keywords:** Rocuronium, Vecuronium, Motor endplate, Neuromuscular junction, Diaphragm

## Abstract

Rocuronium and vecuronium, two non-depolarizing neuromuscular blockers, have been widely used in surgery procedures. However, their electrophysiological properties need to be more widely explored. We examined the effects of rocuronium and vecuronium on initial rundown of endplate potential amplitudes in the non-uniform stretched muscle preparation of the rat isolated phrenic nerve diaphragm. More specifically, the endplate potentials were recorded with one microelectrode from a single endplate. The effects of rocuronium or vecuronium each at 4 concentrations (0.5 ×, l ×, 2 ×, 4 × EC95; EC95 = concentration of the drug required to produce the inhibitory effect by 95%) on the amplitude of endplate potentials and its rundown were observed. Treatment of the isolated rat phrenic nerve-diaphragm preparation with rocuronium (2.5–20 μg/ml) or vecuronium (0.5–4 μg/ml) decreased the amplitude of endplate potentials and inhibited its rundown in a concentration-dependent manner. At the concentration (2.5 μg/ml for rocuronium and 0.5 μg/ml for vecuronium) that did not alter the endplate potential amplitude, the onset of reduced endplate potential rundown was 3 and 5 min after administration of rocuronium or vecuronium, respectively. The results suggest that rocuronium and vecuronium block the neuromuscular junction presynaptically and that rocuronium does it faster than vecuronium.

## Background

The isolated phrenic nerve diaphragm preparation is the most frequently used model for studying electric neurotransmission of the neuromuscular junction, which is one of the most widely studied synapses. The junction consists of three distinct parts: the presynaptic part, which is the distal nerve terminal, the synaptic cleft, and the postsynaptic part, which is a part of the muscle membrane (Fagerlund & Eriksson 2009). Acetylcholine (ACh) is the primary neurotransmitter that is synthesized, stored and released by the nerve terminal. The transmission from nerve to muscle is completed via the release of ACh from presynapses and activation of ligand-gated, fast-acting nicotinic ACh receptors (nAChRs) on postsynapses.

Rocuronium and vecuronium, two non-depolarizing neuromuscular blockers and competitive nAChR antagonists, have been widely used in surgical procedures. While both drugs are monoquaternary aminosteroids and share a similar structure, rocuronium is much less potent, with the ED95 of 0.36 mg/kg, compared to the ED95 of 0.05 mg/kg for vecuronium (Foldes et al. 1991; Xue et al. 1998). The lack of potency is considered to be an important factor in determining the onset of neuromuscular blocking (Bowman et al. 1988). Agents with less potency are more likely to achieve a rapid onset of action. Rocuronium produces the maximum effect within 2 min, which is much more rapid than any other non-depolarizing relaxant (Bevan 1994; Nava-Ocampo et al. 2006). As for rocuronium, nearly all neuromuscular blocking molecules are bound to the postsynaptic nAChRs at the neuromuscular junction. Rocuronium also binds to the presynaptic N-cholinergic receptor at the neuromuscular junction (Meistelman et al. 1992), leading to presynaptic inhibition (Schwarz et al. 1985). This is thought to contribute to the rapid onset of rocuronium’s action (Feldman & Hood 1994). However, electrophysiological evidence is still lacking for the pharmacological properties of non-depolarizing neuromuscular blockers with rapid onset of actions. In the present study, we aimed to compare the effects of rocuronium and vecuronium on initial rundown of the amplitude of endplate potentials (EPPs) in the non-uniform stretched muscle preparation of the isolated rat phrenic nerve diaphragm, in order to determine the inhibitory effect of both drugs on presynaptic transmission.

## Methods

Male and female adult Wistar rats (the Animal Center of the Academy of Military Medical Sciences, Beijing, China), weighing 160–220 g, were used for the experiments. The left side of the diaphragm with the phrenic nerve was dissected from the rat following the procedures described previously (Bulbring 1997). The phrenic nerve diaphragm was kept in the Tyrode's solution with 95% O_2_ and 5% CO_2_. The shear diaphragm (2 × 2 cm) was prepared and made into the non-uniform stretched muscle preparation. Samples were placed in the water bath, which was placed on the working platform within the network mask. The phrenic nerve was put in the hook of the stimulated electrodes; the distance between the electrodes was 1 mm. Phrenic nerves were stimulated with 8 V super square wave stimulation with pulse width of 10–20 μs via the SEN-3201 stimulator connected to the SS-102 J Isolator (NihonKohden Corporation, Japan). The position of the diaphragm with very slight contraction was located and the motor endplates around the nerve fibers stretching into the diaphragm were identified using the dissecting microscope. All the animal experiments were performed following the procedures approved by the Animal Care and Use Committee of the Academy of Military Medical Sciences, Beijing, China, and the National Institute of Health Guide for the Care and Use of Laboratory Animals (NIH Publications No. 80-23, revised 1996).

Signals were outputted from the 10-Vm connector of the amplifier to the VC-10 oscilloscope (NihonKohden Corporation, Japan) (average voltage height = 10). Resting membrane potential was shown in the amplifier Vm. The miniature EPP (MEPP) and EPP, which were amplified using the VC-10 AC (sensitivity 10 mV/cm) and DC amplification (sensitivity 0.1 V/cm), respectively, were outputted and stored in the DTC-75ES tape recorder (Nihon Kohden Corporation, Japan). The signals were collected and saved in the computer from the ISC-67 module (A/D; 16-channel, 12-bit, 1 μs) converting board (RC Electronics Corporation, USA), with the collecting frequency 10 ~ 20 kHz. Signal acquisition and analysis were performed using the Computerscope-PHY system (RC Electronics Corporation, USA).

Rocuronium and vecuronium (N.V. Organon, Oss, The Netherlands) were diluted with the Tyrode's solution. The effects of rocuronium and vecuronium at the concentrations of 0.5 ×, l ×, 2 ×, 4 × EC95 (EC95 = concentration of the drug required to produce the inhibitory effect by 95%) on the EPP amplitude and its initial rundown (i.e., the decrease in the size of the EPP) were examined and compared with the vehicle (the Tyrode’s solution) control. More specifically, each drug was tested in the following groups of concentrations: 0 (vehicle control), 2.5, 5, 10, and 20 μg/ml for rocuronium and 0, 0.5, 1, 2, and 4 μg/ml for vecuronium. All the drug concentrations were identified as the final concentrations in the 20-ml bath. The data was recorded at three time points for each drug after administration, i.e., 1, 3, and 5 min after rocuronium and 3, 5, and 10 min after vecuronium. The concentrations and time points were determined based on our preliminary study and studies published elsewhere, which demonstrate that rocuronium is less potent and has about a half of the onset time relative to vecuronium (Xue et al. 1998; Chatrath et al. 2010). The rundown rate is the percentage of the difference between the first quantum content of EPP and the 11–55 average quantum content of EPP divided by the first quantum content of EPP in the trains of EPPs. Data collected and analyzed by the Computerscope-PHY were graphed using Origin 2.94.

### Data analysis

All values are expressed as means ± SEM. The data were analyzed using one-way analysis of variance (ANOVA) followed by *post hoc* Student’s *t* tests. *P* < 0.05 was considered statistically significant.

## Results

**Figure 1:**
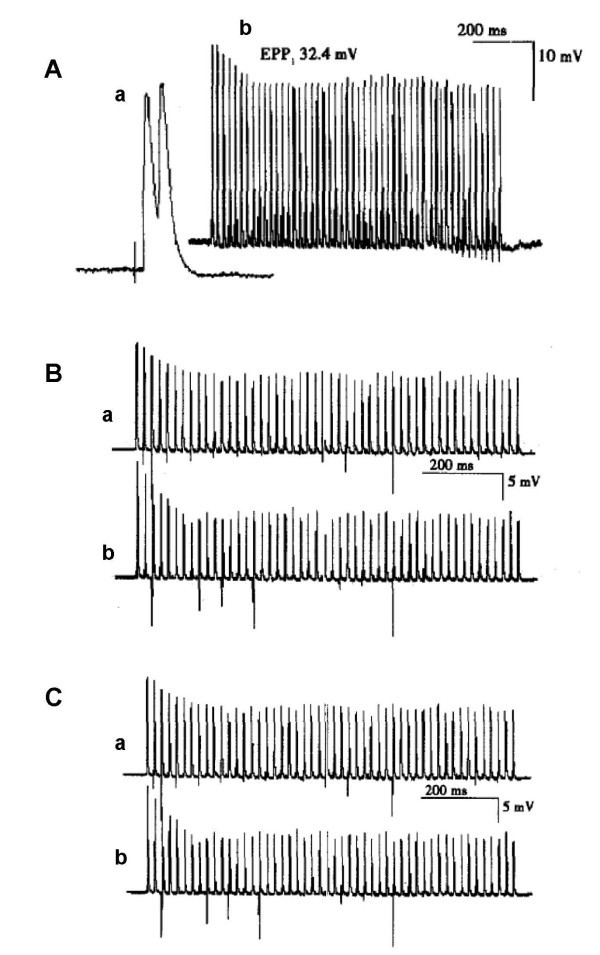
**Effects of rocuronium and vecuronium on the rundown rate of trains of endplate potentials (EPPs) in the isolated phrenic nerve diaphragm preparation of rats.** (**A**) Examples of the single normal EPP (a) and trains of EPPs (b) in the isolated phrenic nerve diaphragm preparation. The second wave of the normal EPP was higher than the first wave (a). In the trains of EPPs (b), the amplitude of EPP1 was 32.4 mV; the average amplitude of EPP11-50 was 27.8 mV; the decay rate (R) = [1−(EPP11-50 average quantum content/EPP1 quantum content)] × 100% = 74%. n = 2 (a) or 50 (b). (**B**) The effect of rocuronium (2.5 μg/ml) on the rundown rate of trains of EPPs at 3 min after drug administration. (a) Normal control; (b) post-treatment of rocuronium. (**C**) The effect of vecuronium (0.5 μg/ml) on the rundown rate of trains of EPPs at 5 min after drug administration. (a) Normal control; (b) post-treatment of vecuronium.

Normal EPPs and trains of EPPs are shown in Figure 1A. The EPP amplitudes were not different among the concentration groups before treatment with rocuronium or vecuronium (Tables 1 and 2), suggesting similar EPP baselines before drug treatment. Rocuronium (2.5–20 μg/ml) decreased the amplitude of EPPs in the non-uniform stretched muscle preparation in a concentration-dependent manner. Specifically, while rocuronium at 2.5 μg/ml did not affect the EPP amplitude, it reduced the EPP amplitude at the concentration of 5 μg/ml at 3 and 5 min after drug administration (*P* < 0.05 and *P* < 0.01, respectively); at higher concentrations of 10 and 20 μg/ml, rocuronium decreased the EPP amplitude at all the three time points (i.e., 1, 3, and 5 min after drug administration; *P <* 0.05 or *P* < 0.01; Table 1).

**Table 1:** **Effects of rocuronium on the amplitude of endplate potentials (EPPs) in the isolated rat phrenic nerve diaphragm preparation at different time points after treatment**

Rocuronium (μg/ml)	EPP (mV) before treatment	EPP (mV) at different times after treatment		
		1 min	3 min	5 min
Control	32.4 ± 3.3	32.2 ± 3.4	32.2 ± 3.3	31.4 ± 3.5
2.5	32.6 ± 3.6	32.3 ± 3.4	32.1 ± 3.5	31.2 ± 3.3
5.0	33.5 ± 3.4	32.4 ± 3.3	22.4 ± 2.9^*#^	14.6 ± 2.1^**##^
10.0	34.8 ± 4.3	27.2 ± 2.5^**^	16.4 ± 1.3^**##^	12.4 ± 1.3^**##^
20.0	33.2 ± 3.1	22.4 ± 2.3^*#^	12.4 ± 2.3^*#^	9.4 ± 1.1^**##^

^*^P < 0.05, ^**^P < 0.01, compared with the corresponding group without rocuronium; ^#^*P* < 0.05, ^##^*P* < 0.01, compared with the control at the same time point; n = 5. Data shown are means ± SEM.

**Table 2:** **Effects of vecuronium on the amplitude of endplate potentials (EPPs) in the isolated rat phrenic nerve diaphragm preparation at different time points after treatment**

Vecuronium (μg/ml)	EPP (mV) before administration	EPP (mV) at different times after treatment		
		3 min	5 min	10 min
Control	32.4 ± 3.3	32.2 ± 3.3	31.4 ± 3.2	31.2 ± 2.9
0.5	32.3 ± 3.4	31.7 ± 2.9	30.6 ± 3.3	29.7 ± 2.2
1.0	31.5 ± 3.2	27.4 ± 2.7	22.4 ± 2.8^*^	16.4 ± 2.1^**##^
2.0	33.1 ± 3.4	22.4 ± 3.3^*#^	14.4 ± 1.4^**##^	12.4 ± 1.3^**##^
4.0	32.7 ± 3.1	18.4 ± 3.3^**##^	12.4 ± 1.5^**##^	8.4 ± 1.3^**##^

^*^P < 0.05, ^**^P < 0.01, compared with the corresponding group without vecuronium; ^#^*P* < 0.05, ^##^*P* < 0.01, compared with the control at the same time point; n = 5. Data shown are means ± SEM.

Similar to rocuronium, vecuronium at 0.5–4 μg/ml also decreased the EPP amplitude in the non-uniform stretched muscle preparation in a concentration-dependent manner. More specifically, while vecuronium at 0.5 μg/ml did not affect the EPP amplitude compared to the control, it reduced the EPP amplitude at 1 μg/ml at 5 and 10 min after drug administration (*P* < 0.05 and *P* < 0.01, respectively; Table 2). At the higher concentrations of 2 and 4 μg/ml, vecuronium decreased the EPP amplitude at all the three time points (i.e., 3, 5, and 10 min after drug administration (*P* < 0.05 or *P* < 0.01).

To compare the onset and potency of the actions, we examined the rundown rates of EPP amplitudes at 3 and 5 min after treatment with rocuronium or vecuronium, each at the concentration that did not alter the EPP amplitude (i.e., 2.5 μg/ml for rocuronium and 0.5 μg/ml for vecuronium). Treatment with either of the drugs at the low concentration decreased initial rundown rates of EPP trains. Rocuronium had slightly greater efficacy at 2.5 μg/ml 3 min after administration relative to vecuronium at 0.5 μg/ml 5 min after administration (Figure 1B, C). In addition, compared to vecuronium, rocuronium generated trains of EPP rundown earlier, while the potency is smaller (2.5 μg/ml vs. 0.5 μg/ml required respectively to generate the maximum effect; Table 3).

**Table 3:** **Changes in string initial rundown rates of endplate potentials (EPPs) before and after treatment of the isolated rat phrenic nerve diaphragm preparation with rocuronium or vecuronium**

Drug (μg/ml)	Before treatment			3 min after treatment			5 min after treatment		
	M1	MX	R (%)	M1	MX	R (%)	M1	MX	R (%)
Rocuronium 2.5	46 ± 11	34 ± 10	26	46 ± 10	28 ± 10	40*	45 ± 12	26 ± 10*	42*
Vecuronium 0.5	46 ± 11	33 ± 11	28	46 ± 11	31 ± 10	33	45 ± 12	29 ± 11*	37*

^*^P < 0.05, compared with the corresponding group without drug treatment; n = 5. M1, the first quantum content of EPP; MX, the 11 to 55 average quantum content of EPP; R, the rundown rate. M = Vc/q; Vc is the corrected amplitude of EPP and q is the quantum size of the MEPP. Data shown are means ± SEM.

## Discussion

The neuromuscular junction is a typical class of peripheral cholinergic synapses. Under normal circumstances, nerve stimulation produces presynaptic release of a large number of ACh, leading to a postsynaptic endplate potential and a subsequent mechanical contraction via muscle action potentials. In physiological conditions, a bunch of nerve impulses (about 50 Hz) stimulates release of a large amount of ACh from presynaptic nerve endings; as the neurotransmitter, ACh produces trains of postsynaptic membrane potentials and endplate action potentials, leading to tonic muscle contraction. ACh in the synaptic cleft (gap) is rapidly hydrolyzed by AChE, causing a rapid decrease in the ACh level to that before ACh release and subsequently terminates the role of ACh timely and effectively. This rapidly repolarizes the postsynaptic membrane potential for responding to the next release of ACh (Martin 2005). The attenuation of 50-Hz or 100-Hz tonic stimulation is resulted from blockade of presynaptic receptors. There are two types of vesicles for presynaptic release of ACh: immediate release vesicles and storage vesicles. Under the tonic stimulation of 50 Hz or 100 Hz, ACh immediate-release vesicles are empty while ACh is continuously transported from storage vesicles, leading to decreases in ACh levels, EPP amplitudes, and rundown rates of EPP trains. These in turn result in feedback regulation of ACh, which maintains normal physiological function by preventing EPP rates from further decreases (Bowman 1990). In the present study, we examined the presynaptic effects of rocuronium and vecuronium based on the initial attenuation of trains of EPPs caused by 50-Hz tonic stimulation.

In the neuromuscular junction, ACh in the synaptic cleft can play a positive or negative role in the feedback regulation of ACh release from nerve terminals via the action on presynaptic nAChRs (Fagerlund & Eriksson 2009; Wessler 1989). More specifically, ACh at low or physiological concentrations plays a positive feedback role in ACh release, leading to increased immediate-release vesicles transferred from storage vesicles in the nerve endings. The positive feedback mechanism allows nerve-muscle tissues to adapt to high-frequency stimulation (> 1 Hz) (Bowman 1980). In contrast, ACh at high concentrations plays a negative feedback role in ACh release, leading to depolarization of the nerve terminals. These two feedback mechanisms may affect the onset of actions of non-depolarizing muscle relaxants to a certain extent. The non-uniform stretched muscle preparation from the isolated rat phrenic nerve diaphragm is used to record relatively high rates of EPPs and miniature EPPs (Zou & Liu 1988). Using this approach, we recorded an average of 30 mV of EPP.

There are two types of rundown: intratrain rundown (reduction in the EPP size from one train to the next), which is frequency-dependent, and intertrain rundown (reduction in the EPP size within a train), which is relatively independent of stimulation frequency during intermittent stimulation (Moyer & van Lunteren 1999). The initial rundown (intratrain rundown) of the EPP amplitude usually decreases quickly within a train but recovers almost completely from train to train during intermittent stimulation. In the present study, administration of rocuronium or vecuronium decreased both intratrain rundown and intertrain rundown of EPPs.

While most neuromuscular blocking agents act on postsynaptic nicotinic receptors at the neuromuscular junction, several lines of evidence also suggest a presynaptic role of rocuronium and vecuronium. The impact of rocuronium on the EPP initial rundown is related to the frequency of stimulation: the higher stimulus frequency, the more initial rundown. This feature indicates that rocuronium may have a potent presynaptic effect (Feldman & Khaw 1995). This is supported by the presynaptic effect of vecuronium (Schwarz et al. 1985). In addition, using the isolated human forearm, England and co-workers have demonstrated that rocuronium produces non-depolarizing blockade in a manner different from other non-depolarizing muscle relaxants in terms of onset and/or recovery (England et al. 1996), suggesting that rocuronium exerts a presynaptic effect. Further, more directly, rocuronium binds to the presynaptic N-cholinergic receptors at the neuromuscular junction, leading to presynaptic inhibition (Meistelman et al. 1992; Feldman & Hood 1994).

Both rocuronium and vecuronium enhances ACh release from presynapses. Compared to vecuronium, rocuronium acts on EPP amplitudes with slightly greater efficacy and faster onset, but smaller potency. It is speculated that rocuronium has faster and greater presynaptic inhibition (Li & Zhang 2004). The inhibitory effects of the drugs on the initial rundown of EPP amplitudes indicate that presynaptic inhibition by rocuronium may be related to the direct effect on presynaptic nAChRs, leading to reduced transport of neurotransmitters in presynapses.

Of note, sensitivity to rocuronium and/or vecuronium may vary in certain medical conditions interfering with neurotransmission at the neuromuscular junction. For instance, patients with myasthenia gravis, an autoimmune disorder involving the destruction of AChRs at the postsynaptic membrane at the neuromuscular junction, are hypersensitive to nondepolarizing neuromuscular blocking drugs such as vecuronium, which can make the characteristic symptoms such as fatigue worse (Itoh et al. 2002). Similarly, patients with neuromyotonia, a disorder of hyperexcitability of the peripheral nerve and characterized by both myokymic and neuromyotonic discharges, also exhibit increased sensitivity to the neuromuscular effects of rocuronium (Ginsburg et al. 2009). Thus, lower doses of rocuronium/vecuronium should be administered for anesthesia in patients with these disorders.

In conclusions, both rocuronium and vecuronium enhance initial rundown of EPP amplitudes in the isolated phrenic nerve diaphragm preparation of rats, suggesting that both drugs produce significant presynaptic inhibition. Compared to vecuronium, rocuronium is slightly greater in efficacy and faster in onset, but smaller in potency of blocking the neuromuscular junction.
